# Randomized Controlled Clinical Trial Comparing Screw‐Retained Monolithic Zirconia Implant Crowns on Standardized Titanium Base Versus Customized Titanium Abutments– 3‐Years Data

**DOI:** 10.1111/clr.70165

**Published:** 2026-08-06

**Authors:** Jenni Hjerppe, Stephanie Wenk, Shunsuke Fukuba, Sven Mühlemann, Ronald E. Jung, Daniel S. Thoma

**Affiliations:** ^1^ Clinic of Reconstructive Dentistry, Center for Dental Medicine University of Zurich Zurich Switzerland; ^2^ Department of Regenerative and Reconstructive Dental Medicine The Institute of Science Tokyo Tokyo Japan; ^3^ Private Dental Office Zürich Switzerland; ^4^ Department of Periodontology, Research Institute for Periodontal Regeneration Yonsei University College of Dentistry Seoul South Korea

**Keywords:** clinical trials, crowns, dental implants, patient satisfaction, prosthodontics, survival, titanium, zirconium oxide

## Abstract

**Objectives:**

To assess 3‐year interim results of implant‐supported monolithic zirconia single crowns on standardized titanium base vs. customized titanium abutments.

**Material and Methods:**

Patients in need of posterior implant were recruited. After implant healing time, patients were randomly allocated to: Ti‐Base (*n* = 30) with a monolithic zirconia crown on a standardized Ti‐Base abutment or Custom (*n* = 30) with a monolithic zirconia crown on a customized titanium abutment. Implant crowns were evaluated at Baseline (BL), 1‐year (1Y‐FU), and 3 years (3Y‐FU) follow‐ups for marginal bone level changes (MBL), survival, biological and technical outcomes, and PROMs. Chi‐squared and Fisher's exact test were used to assess association between categorical variables, Mann–Whitney's test to compare the distribution of parameters between groups, with a significance level of 5*%* (α = 0.05).

**Results:**

Fifty‐one patients (23 Custom, 28 Ti‐Base) attended 3Y‐FU visit. No implant crowns were lost. Similar median MBL changes from BL to 3Y‐FU and comparable biological outcomes were observed in both groups (*p* > 0.05). No major technical complications were observed, but one minor chipping of Ti‐Base zirconia crown was recorded at 3Y‐FU, resulting in a technical complication rate of 3.6% for this group. At the 3Y‐FU, all patients in the Custom group and 26 patients (92.9%) in the Ti‐Base group were very satisfied with the treatment (*p* > 0.05).

**Conclusions:**

Within the limitations of this interim analysis, monolithic zirconia single crowns on either standardized titanium base or customized titanium abutments demonstrated stable marginal bone levels, high survival rates, high patient satisfaction, and no major technical complications during the 3Y follow‐up period.

**Trial Registration:**

https://drks.de/search/en/trial/DRKS00012733

## 1 Introduction

Single‐tooth implant restorations represent a well‐established approach for replacing missing teeth, with the goal of restoring both function and aesthetics (Buser et al. 2012). Traditionally, metal–ceramic implant‐supported restorations have been considered the standard of care in implant prosthodontics due to their favorable mechanical properties (Pjetursson, Valente, et al. 2018). However, disadvantages have been reported, including complex laboratory procedures, the high cost of noble metal alloys, and aesthetic limitations related to potential grayish discoloration at the mucosal margin (Muhlemann et al. 2018, 2022; Pitta et al. 2020).

At the beginning of the digital era, with the introduction of computer‐aided design/computer‐aided manufacturing (CAD/CAM) workflows, centrally manufactured customized zirconia and titanium abutments were introduced (Marchack 1996; Sailer et al. 2009). The aim was to fabricate anatomically tailored abutments to support each patient's individual clinical situation while increasing efficiency and enabling novel fabrication methods for dental technicians, shifting from casting to milling (Joda et al. 2016). Customized zirconia abutments were particularly intended for anterior restorations due to their favorable optical properties. Nevertheless, their clinical reliability remains under discussion, as long‐term clinical studies have reported an increased risk of abutment fractures and irreversible damage to the implant–abutment interface (Kraus et al. 2024; Pjetursson, Zarauz, et al. 2018). In contrast, customized titanium abutments have demonstrated a 100% survival rate over a 13‐year follow‐up period (Humm et al. 2023).

Advances in restorative materials and digital workflows have led to the introduction of prefabricated, standardized titanium base (Ti‐Base) abutments. These components enable standardized implant–abutment connections and are designed to be adhesively bonded to CAD/CAM‐fabricated monolithic restorations (Joda et al. 2017; Zhang et al. 2024). Ti‐Base abutments aim to combine efficient workflows with reproducibility, mechanical strength, and acceptable esthetic outcomes (Hjerppe et al. 2022; Muhlemann et al. 2018, 2022; Pitta, Hjerppe, et al. 2021; Zhang et al. 2024). However, in vitro studies have reported fractures and bonding failures at the restoration–Ti‐Base abutment interface (Calderon et al. 2022; Ioannidis et al. 2020; Pitta, Hjerppe, et al. 2021; Schafer et al. 2025). Such failures may result in prosthetic decementation or micromovement, potentially compromising peri‐implant health due to microbial infiltration at the microgap between the Ti‐Base abutment and the crown (Nascimento et al. 2015; Sasada and Cochran 2017; Teixeira et al. 2011).

Prospective clinical studies with 3–5 years follow‐up time have reported 100% survival rates and few complications for Ti‐Base‐supported zirconia crowns in posterior regions (Beck et al. 2024; Derksen and Wismeijer 2023; Zumstein et al. 2023). The documented technical complications included decementations, screw loosening, and fracture of a Ti‐Base abutment. Despite these promising short‐term outcomes, concerns remain regarding the technical, biological, and esthetic performance of Ti‐Base‐supported restorations. Furthermore, no studies to date have compared clinical outcomes of these with monolithic zirconia crowns supported by customized titanium abutments.

Therefore, the aim of this randomized controlled clinical trial was to evaluate the interim 3‐year outcomes regarding marginal bone levels, technical and biological complications, and patient‐reported outcome measures (PROMs) of implant‐supported monolithic zirconia single crowns using titanium base abutments, comparing those to customized titanium abutments. The null hypothesis was that both restoration types would yield similar results.

## 2 Materials and Methods

### 2.1 Study Design

This study was designed as a parallel‐arm randomized controlled clinical trial with two study groups on prosthetic level and was conducted at the Clinic of Reconstructive Dentistry, University of Zürich, Switzerland. The study protocol was approved by the local ethics committee (Kantonale Ethikkommission, Kanton Zürich, BASEC‐Nr. 2017–01401), and the trial was registered at the German Clinical Trials Register (DRKS00012733). All patients provided written informed consent prior to any study procedures. The patients were treated in accordance with ISO EN 14155 and the Declaration of Helsinki. This article follows the CONSORT reporting guidelines (Moher et al. 2010).

### 2.2 Study Population

Patients were recruited for the study from December 2017 to June 2019. The study population consisted of partially edentulous patients requiring a single implant in the maxillary or mandibular premolar or molar region. The participants fulfilling the following criteria were included to the study:

- Signed informed consent form
- Age > 18 years
- Good general health
- Requirement for one implant in a healed posterior site (premolar and molar region)
- Need for a single implant‐supported crown in the non‐aesthetic area
- Presence of an antagonist
- Presence of at least one neighboring tooth or implant

The presence of any of the following criteria was leading to exclusion of the participant:

- Women who were pregnant or breastfeeding at the time of the implant placement
- Known or suspected noncompliance, drug or alcohol use
- Inability to follow study procedures due to language barriers, psychological disorders, dementia, or other conditions
- Heavy smoking (> 10 cigarettes per day)
- Poor oral hygiene after hygienic phase treatment (Plaque Index > 30%)
- Active periodontal disease

### 2.3 Implant Placement

After the patients were successfully recruited, delayed or late implant placement was performed, corresponding to 3–6 months or > 6 months after extraction, respectively. A crestal incision was made along the alveolar ridge, and a sulcular incision was performed on the adjacent teeth, with a releasing incision, if necessary. A full‐thickness muco‐periosteal flap was elevated, and the implant was placed according to the manufacturer's guidelines (Osseospeed EV, Astra Tech Implant System, Dentsply Sirona Implants, Mölndal, Sweden).

When indicated, guided bone regeneration was performed using deproteinized bovine bone mineral (DBBM) (Bio‐Oss, Geistlich Pharma AG, Switzerland) and a resorbable collagen membrane (Bio‐Gide, Geistlich Pharma AG, Switzerland). The membrane was perforated to accommodate a healing abutment, and all the implants were left to heal transmucosally. Non‐resorbable sutures (Dafilon 5–0, Braun) were used to achieve tension‐free flap closure. The study procedures have been partially described in two sub‐analyses of this randomized controlled clinical trial (Muhlemann et al. 2022; Zuercher et al. 2024).

### 2.4 Randomization

The present study was originally designed as a prospective randomized controlled clinical trial with four study arms and a randomization on the implant and on the prosthetic level. The first randomization will take place at the timepoint of implant placement and the patients will be allocated to two treatment groups for the buccal dehiscence: (1) Test group: Spontaneous healing of bony defect (no guided bone regeneration, GBR performed, *n* = 30) and (2) Control group GBR: guided bone regeneration with bone substitute material (Bio‐Oss granules) and collagen membrane (Bio‐Gide), *n* = 30. Then at the timepoint of final impression the patients will be randomly allocated to two prosthetic groups: (1) Test Group “Ti Base” with monolithic zirconia crown bonded to Atlantis TitaniumBase EV abutment, *n* = 30 and (2) control group “Custom” with monolithic zirconia crown bonded to Atlantis Titanium Abutment EV, *n* = 30. This would result in four study groups (*n* = 15): (1) Spontaneous healing and Ti‐Base, (2) Spontaneous healing and Custom Abutment, (3) GBR and Ti Base, and (4) GBR and Custom Abutment.

At the timepoint of implant placement, in case of a buccal dehiscence defect (≤ 5 mm) the group on implant level was drawn with a sealed envelope (Zuercher et al. 2024). As 16 patients out of 60 were identified with buccal dehiscence defect half of them were randomized to test group (spontaneous healing, *n* = 8) and half of them to control group (guided bone regeneration with bone substitute material and collagen membrane, *n* = 8). The results of this sub analysis with buccal dehiscence defects have been published before (Zuercher et al. 2024).

After 3–6 months of healing, the final impression for the study crowns was taken. At this visit, the patients were randomly assigned to one of two prosthetic crown groups. Also, the patients previously randomized on implant level were now randomly assigned to one of the two prosthetic groups. Randomization was concealed by an independent study using a computer‐generated randomization list (www.sealedevenlope.com). Allocation was performed in a 1:1 ratio in block randomization of four, and sealed envelopes were drawn to assign patients to the study groups:

- Test group (Ti‐Base): Monolithic zirconia crown (Ceramill Zolid + Preshades, Ammann Girrbach) bonded to Ti‐Base abutment (Titaniumbase EV, Dentsply Sirona).
- Control group (Custom): Monolithic zirconia crown (ZPEX, Tosoch Inc., USA; Atlantis crown, Dentsply Sirona) bonded to customized titanium abutment (Atlantis Titanium abutment EV, CustomBase solution, Dentsply Sirona).

### 2.5 Prosthetic Procedures

The detailed manufacturing processes of the study crowns have been previously described (Muhlemann et al. 2022). Briefly the procedure was as follows: For the Custom group, a full arch optical impression was taken using an intraoral scanner (Trios 3, Software 1.4.7.0, 3Shape) with a screw‐retained scan body attached to the study implant (Elos Accurate IO Scan body, Elos Medtech). Additionally, a full arch scan of the antagonist jaw and bite registration in maximal intercuspidation were completed. The scans were sent to the manufacturer for computer‐aided design (CAD) and centralized fabrication of the customized titanium abutment (Atlantis, CustomBase solution, Dentsply Sirona) and the full‐contour monolithic zirconia crown (Atlantis Crown, Dentsply Sirona). The abutments were individually designed for each patient, with gingival height and dimensions determined by the thickness of the peri‐implant mucosa and the location of anatomical landmarks (bone crest and adjacent teeth) in the implant site. The abutment margin was placed approximately 1 mm below the mucosal margin.

For the Ti‐Base group, a conventional full‐arch impression was taken with polyether impression material (Permadyne, 3 M) using a screw retained impression post (Implant Pick‐up EV, Astra tech, Dentsply Sirona). Hydrocolloid material (Cavex Alginate, Cavex) was used for the antagonist, and silicone material (Preciform, Merz Dental) was used for occlusal registration. Conventional stone models were cast, and a monolithic zirconia crown (Ceramill Zolid HT+ preshades, Ammann Girrbach) on a titanium base abutment (Titaniumbase EV, Gingival height 1 mm, Dentsply Sirona) was fabricated in a local dental laboratory.

During the try‐in visit anatomical form, proximal mesial and distal contacts, occlusion and color were assessed using modified United States Public Health Services (USPHS) criteria (Table 1). Additionally, the need for chairside adjustments was recorded. These results have been published as a sub‐analysis of the present study investigating the different fabrication workflows (Muhlemann et al. 2022). After a try‐in visit, the titanium abutments and cementation surface of zirconia crowns in both groups were air‐borne particle abraded with 50 μm aluminum oxide for 10 s with 1 bar pressure at 1 cm distance (Rocatec Plus, 3 M) and cleaned with a steam cleaner. A bonding agent containing silane and MDP‐monomer (Monobond Plus, Ivoclar Vivadent) was applied on cementation surfaces of the abutments and crowns for 60 s. The abutments were cemented with self‐curing luting composite (Multilink Hybrid Abutment HO, Ivoclar Vivadent).

**TABLE 1:** Technical parameters evaluated with modified USPHS criteria.

Criteria	A	B	C	D
Patient's satisfaction	Very satisfied, no complaints	Critics regarding esthetics/chewing comfort etc., short‐term complaints after treatment	Unsatisfied, constant complaints, but tolerable	Completely unsatisfied, unbearable complaints
Marginal adaptation crown unit	No probe catch	Slight probe catch, but no gap	Gap	New crown is needed
Veneering fracture	No fracture	Chipping (localized), but polishing/contouring possible	Chipping down the framework, multiple chipping, polishing/contouring not possible	New crown is needed
Abutment fracture	No fracture	—	—	Fracture of abutment
Anatomical form	Ideal anatomical shape	Slightly over‐ or under‐contoured	Highly over‐ or under‐contoured	New crown is needed
Proximal contact distal	Good proximal contact	Weak proximal contact	Open proximal contact	—
Proximal contact mesial	Good proximal contact	Weak proximal contact	Open proximal contact	—
Occlusion	Normal occlusal contact, no balancing contact	Increased occlusal contact, balancing contact	No occlusal contact, hyper balancing contact	—
Occlusal wear	No occlusal wear on crown	Slight occlusal wear, Ø < 2 mm on crown	High occlusal wear, Ø > 2 mm on crown	—
Color	No deviation in color and translucency between crown and dentition	Deviation between crown and dentition within natural deviation	Deviation between crown and dentition outside natural deviation	—
X‐ray	Cement gap not visible in x‐ray	Cement gap slightly visible in x‐ray	Cement gap highly visible in x‐ray	Intolerable cement gap

The crowns were inserted onto the implants with a torque of 25 Ncm, according to the manufacturer's instructions. The screw access channel was sealed with PTFE tape (Henry Schein Dental). The screw access hole was closed with a composite filling material (Monobond Plus, Heliobond; Ivoclar Vivadent; CeramX Spectra composite, Dentsply Sirona).

### 2.6 Clinical Examination and Outcome Measures

Two calibrated operators performed all clinical examinations at baseline (BL; 1–2 weeks after crown insertion), as well as at the 1‐year (1Y‐FU) and the three‐year (3Y‐FU) follow‐up visits. Operator calibration was conducted through a calibration meeting and practice examinations on pilot patients. Blinding of the operators was not possible due to the type of restoration being identifiable on the radiographic images. Radiographic, technical, and biological outcomes were assessed at BL, 1Y‐FU, and 3Y‐FU visits. Implant survival was recorded according to the criteria of Buser and co‐workers (Buser et al. 2012). At each visit, clinical photographs of the implant crown and the neighboring teeth were taken, along with digital impressions of full dentition (3Shape, Trios).

#### 2.6.1 Radiographic Outcomes

Marginal bone levels (MBL) were measured on the mesial and distal aspects of the study implants at BL, 1Y‐FU, and 3Y‐FU by determining the distance between the implant shoulder and the first bone‐to‐implant contact. The inter‐thread pitch distance (0.6 mm) was used to calibrate apical‐coronal measurements on each radiograph by using open‐source software (ImageJ 1.50i, National Institutes of Health, USA). MBL assessments were performed independently by two examiners (JH and SF). After the calibration meeting, both examiners measured the marginal bone levels on the mesial and distal aspects of the study implants at each timepoint.

#### 2.6.2 Technical Outcomes

Crown survival was recorded at each follow‐up visit and defined as the crown remaining in situ. Technical complications included crown fracture, abutment fracture, and decementation of the crown from the abutment. Additional technical outcomes of the study crowns, as well as patient‐reported outcomes (PROs), were evaluated using modified United States Public Health Services (USPHS) criteria (Table 1) (Bayne and Schmalz 2005).

#### 2.6.3 Biological Outcomes

Peri‐implant and periodontal parameters, including probing pocket depth (PPD), bleeding on probing (BOP), and plaque control record (PCR), were assessed at six sites around the study implants and around the mesial and distal neighboring teeth (Ainamo and Bay 1976; O'Leary et al. 1972). The width of the keratinized mucosa (KM) was measured at the mid buccal aspect of the study implants and the neighboring mesial and distal teeth using a periodontal probe. Mucosal recession was evaluated by measuring the crown height at the mid buccal aspect at each visit, defined as the distance from the mid‐buccal mucosal margin to the incisal edge or occlusal surface of the crown.

Peri‐implant mucositis and peri‐implantitis were assessed according to European Federation of Periodontology (EFP) guidelines. Peri‐implant mucositis was diagnosed when (Herrera et al. 2023; Tonetti et al. 2023):

- Presence of bleeding on more than one spot at a location around the implant or presence of a line of bleeding or profuse bleeding at any location and/or suppuration on gentle probing.
- Absence of bone loss beyond crestal bone level changes resulting from initial bone remodeling

Peri‐implantitis was diagnosed when (Berglundh et al. 2018; Herrera et al. 2023):

- Bleeding and/or suppuration on gentle probing
- Increased PPD compared to previous examinations
- Bone loss beyond crestal bone remodeling, as assessed on periapical radiograph.

### 2.7 Statistical Analysis

The primary outcome of this randomized controlled clinical trial was the radiographic bone level at 5 years. Data for sample size calculation were derived from a 5‐year follow‐up study with a two‐piece (AstraTech ST) implant (Palmer et al. 2000). The statistical parameter considered was the increase in vertical distance from the implant shoulder to the first bone‐to‐implant contact between 1 and 5 years. A bone resorption of 1.5 mm in the first year and 0.2 mm annually thereafter was considered acceptable, whereas a bone loss of 0.5 mm (i.e., 2.8 mm at the 5 years) was deemed clinically relevant (Palmer et al. 2000). The 5‐year study reported a standard deviation for bone loss of 0.46 mm, which was used for the following sample size calculation.

One sample *t*‐test was used to detect bone loss after 5 years: A sample size of 3 achieves 80% power to detect a difference between a null hypothesis mean of 0.0 mm and an alternative mean of 2.3 mm at a two‐sided significance level of 0.05, assuming a standard deviation of 0.46 mm. A two sample *t*‐test was used to detect the difference in the bone loss between the control and the test groups: A sample size of 15 per group achieves 80% power to detect a difference in means of −0.5 mm (control: 2.3 mm, test: 2.8 mm) with a common standard deviation of 0.46 mm using a two‐sided significance level of 0.05. The patients were randomized to surgical study arm in case there was a buccal dehiscence present. At the time point of implant placement, 16 study sites had a buccal defect. This patient population was randomized to GBR and spontaneous healing groups. A sub‐analysis on buccal bone levels of implants with a dehiscence defect treated with or without guided bone regeneration (GBR) using 8 patients per group has been published previously (Zuercher et al. 2024). To account for secondary outcomes (marginal bone level at 3 years, technical complications, crown and abutment survival) and an expected dropout rate of approximately 15%, a total sample size of 30 per group (60 patients altogether) was chosen (Jung et al. 2012).

Statistical analysis consists of a descriptive analysis of categorical variables (absolute and relative frequencies) and continuous variables (mean, standard deviation, range, and median) for the total sample, differentiating by crown type. A Kolmogorov–Smirnov test was conducted to assess if distributions of parameters and differences fit normal patterns, showing significant deviations in most of them. Because of the moderate sample size, a nonparametric approach was considered in the analysis.

Mann–Whitney's test was used to compare the distribution of parameters between groups. Whereas Chi‐squared and Fisher's exact test were used to assess the association between categorical variables. *Significance level* used in the analysis was 5*%* (α = 0.05).

Due to the COVID‐19 pandemic, some patients missed scheduled study visits. When missing data exceeded 10% for a given time point, the last‐observation‐carried‐forward (LOCF) method was applied, carrying forward the last available measurement prior to the missed visit (Shao and Zhong 2003). All analyses were performed following the intention‐to‐treat principle.

In order to evaluate the reliability of marginal bone level measurements between two examiners (JH and SF), the reliability analysis was done with statistical software (SPSS Statistics v 29.0, IBM). Inter‐examiner reliability was evaluated by using the intraclass correlation coefficient (ICC) as an estimate of internal consistency across examiners.

## 3 Results

### 3.1 Demographic Results

This RCT was designed with four study arms. At the timepoint of implant placement 16 study sites out of 60 had a small buccal dehiscence (on average less than 3 mm). Therefore, in eight patients minor GBR was done and eight patients were randomized to spontaneous healing group. By the time point of prosthetic procedures these patients were randomly assigned to prosthetic groups resulting in seven GBR + Ti Base sites, one GBR and Customized site, four Spontaneous + Ti Base sites and four Spontaneous and Custom sites. Due to very small number of sites per group, it was not possible to analyze the data according to originally planned four study arms. Therefore, the results of the buccal defect have been published separately and the present work concentrates on the results in prosthetic level (Zuercher et al. 2024).

The results are presented according to prosthetic study arms: A total of 60 patients were recruited for this study, with 30 patients allocated to each group (Figure 1). One patient was excluded before the surgical or prosthetic study procedures due to protocol deviations, and another was excluded due to early implant loss. The remaining 58 patients were randomized to two groups: 30 patients in the Ti‐Base group and 28 patients in the Custom group.

**FIGURE 1:**
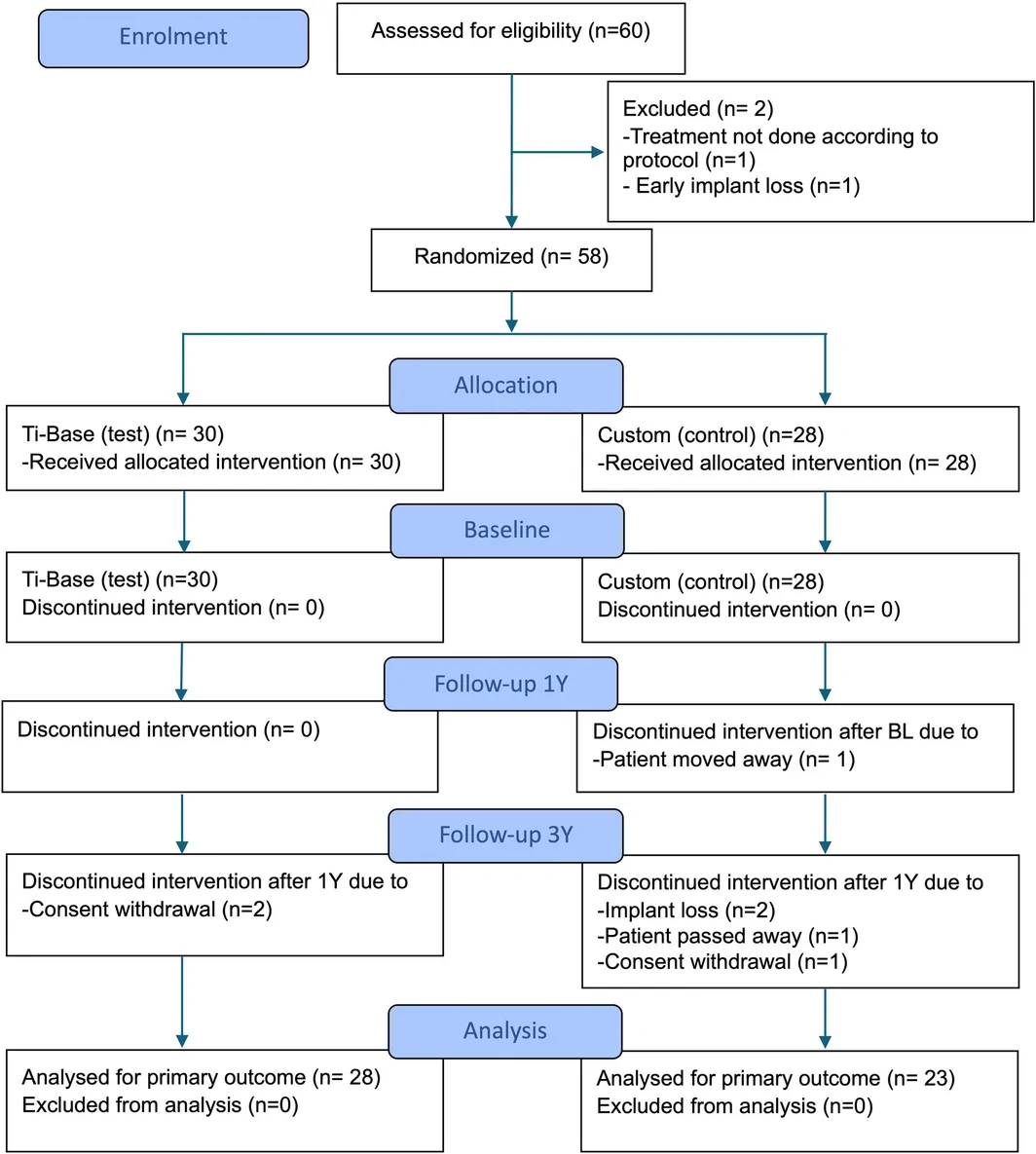
CONSORT 2025 Flowchart illustrating patient recruitment, exclusions, randomization, and follow‐up in the study.

Regarding implant dimensions, two implants were 3.6 mm, 39 were 4.2 mm, 13 were 4.8 mm and four were 5.4 mm in diameter. At the baseline visit, the mean patient age was 57.4 (SD13.8) years, with 27 females and 31 males. Patient demographics at BL are summarized in Table 2. At the 3Y‐FU, a total of 51 patients attended: 23 in the Custom group and 28 in the Ti‐Base group.

**TABLE 2:** Patient and restoration demographics at baseline (BL), presented for each study group (Custom and Ti‐Base). Percentages are calculated for each column, either for the total study population (column “Total”) or per group.

Group	Total	Custom	Ti‐Base
Patients (*n*)	58	28	30
Age			
Years mean (SD)	57.4 (13.8)	58.5 (12.6)	56.3 (15.0)
Gender			
% females (*n*)	46.6 (27)	46.4 (13)	46.7 (14)
% males (*n*)	53.4 (31)	53.6 (15)	53.3 (16)
Restoration			
Jaw			
Maxilla % (*n*)	48.3 (28)	46.4 (13)	50 (15)
Mandible % (*n*)	51.7 (30)	53.6 (15)	50 (15)
Tooth location			
17 % (*n*)	1.7 (1)	0 (0)	3.3 (1)
16 % (*n*)	13.8 (8)	17.9 (5)	10.0 (3)
15 % (*n*)	1.7 (1)	0 (0)	3.3 (1)
14 % (*n*)	10.3 (6)	14.3 (4)	6.7 (2)
24 % (*n*)	1.7 (1)	3.6 (1)	0 (0)
25 % (*n*)	6.9 (4)	7.1 (2)	6.7 (2)
26 % (*n*)	10.3 (6)	0 (0)	20.0 (6)
27 % (*n*)	1.7 (1)	3.6 (1)	0 (0)
37 % (*n*)	1.7 (1)	3.6 (1)	0 (0)
36 % (*n*)	19.0 (11)	14.3 (4)	23.3 (7)
35 % (*n*)	5.2 (3)	7.1 (2)	3.3 (1)
44 % (*n*)	1.7 (1)	3.6 (1)	0 (0)
45 % (*n*)	6.9 (4)	3.6 (1)	10.0 (3)
46 % (*n*)	10.3 (6)	7.1 (2)	13.3 (4)
47 % (*n*)	6.9 (4)	14.3 (4)	0 (0)

### 3.2 Data Imputation

Data imputation method was applied in both groups, as three patients per group had a missed visit during study follow‐up. In the Custom group, the reasons for missing a single visit were pregnancy, COVID‐19 pandemic‐related delays, and the patient living abroad. In the Ti‐Base group, the missed visits were due to pregnancy and two instances of COVID‐19 pandemic‐related delays.

### 3.3 Radiographic Outcomes and Marginal Bone Levels

Marginal bone levels (MBLs) were measured independently by two examiners, and inter‐examiner reliability was assessed using the intraclass correlation coefficient (ICC), demonstrating high agreement between observers (Table 3). Minimal variation in MBLs was observed in both groups over the study period. The median MBL changes from BL to 3Y‐FU were −0.01 mm (IQR −0.32; 0.34) in the Ti‐Base group and −0.09 mm (IQR −0.28; −0.02) in the Custom group (Figure 2). No statistically significant differences were seen in marginal bone levels at between any time points or between the groups (*p* > 0.05).

**TABLE 3:** Inter‐examiner reliability of marginal bone level (MBL) measurements at different time points assessed using the intraclass correlation coefficient (ICC). Time points: Baseline (BL), 1‐year (1Y‐FU), and 3‐year (3Y‐FU).

Inter‐examiner reliability	Parameter	Intraclass correlation coefficient (ICC)	95% CI
	BL mesial	0.845	0.736; 0.909
	BL distal	0.952	0.918; 0.972
	1Y‐FU mesial	0.923	0.868; 0.955
	1Y‐FU distal	0.925	0.871; 0.956
	3Y‐FU mesial	0.963	0.934; 0.979
	3Y‐FU distal	0.983	0.970; 0.990

**FIGURE 2:**
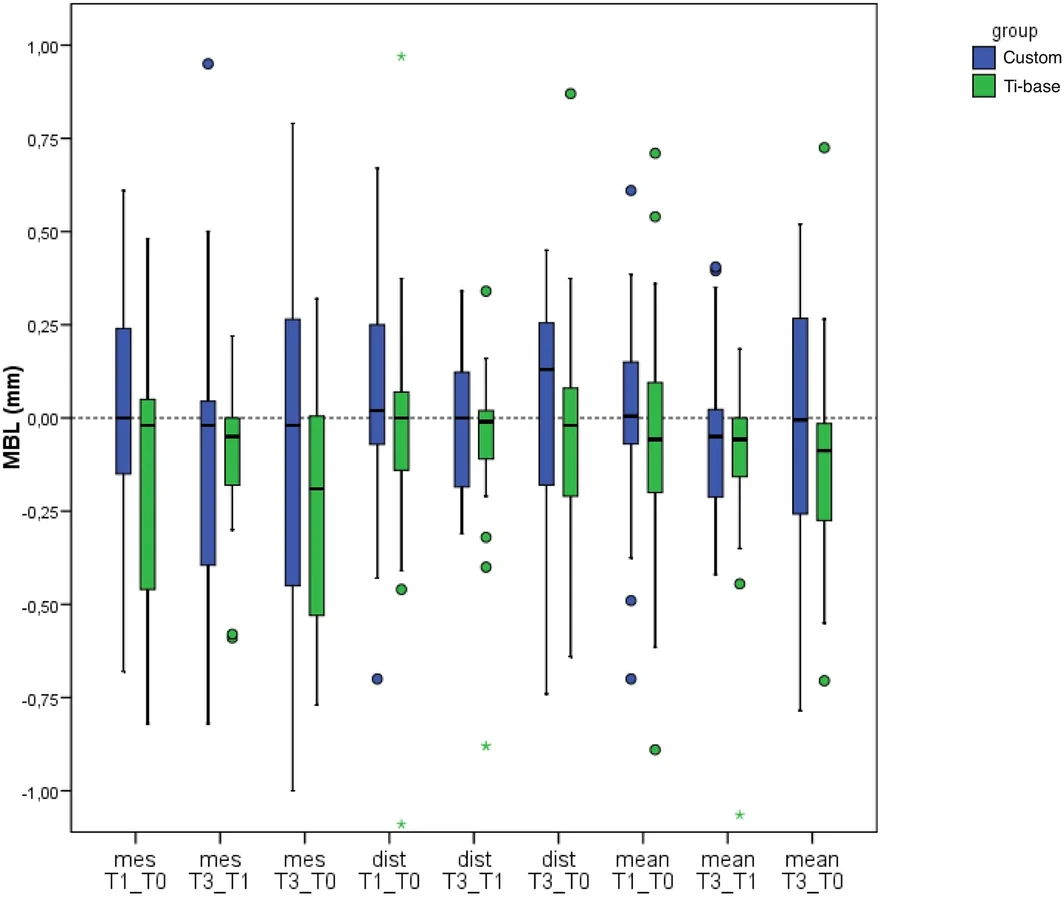
Median variation and interquartile ranges (IQR) of marginal bone levels (MBL, mm) at baseline (BL, T0), 1‐year (1Y‐FU, T1), and 3‐year (3Y‐FU, T2) follow‐up visits. Measurements are reported at the mesial and distal aspect of the study crowns and as the mean of mesial and distal values.

### 3.4 Implant and Crown Survival

During the 3‐year follow‐up, no implants were lost in the Ti‐Base group, whereas two were lost in the Custom group due to loss of osseointegration and implant fracture. Consequently, the implant survival rates were 100% for the Ti‐Base group and 92.9% for the Custom group. However, no significant difference between the groups in terms of implant loss was seen (*p* = 1). No implant crowns were lost in either group during the follow‐up period, resulting in a 100% crown survival rate in both groups. Figure 3 presents clinical documentation of representative cases from each group.

**FIGURE 3:**
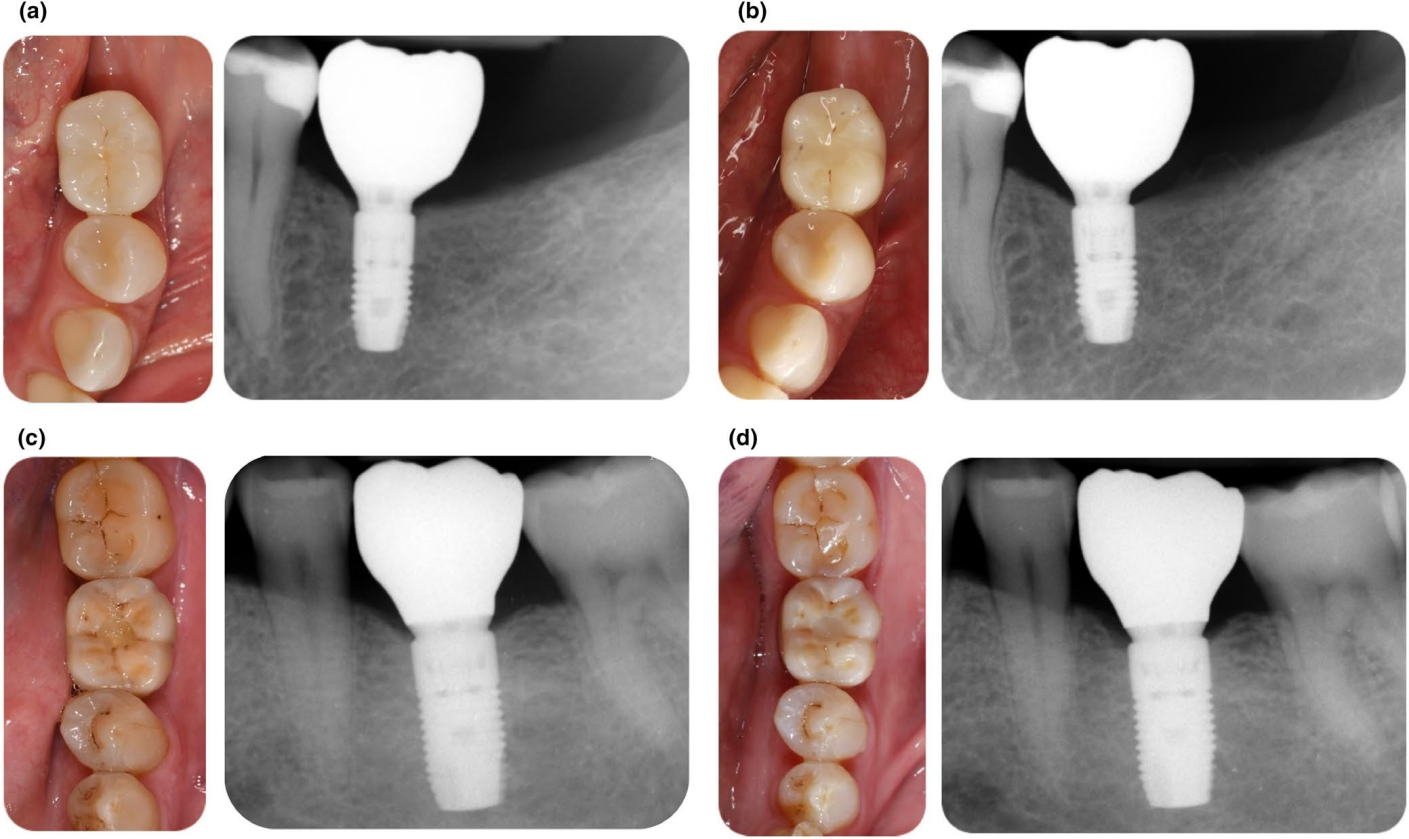
Clinical and radiographic documentation of representative cases from the Custom and Ti‐Base groups at BL and 3‐year follow‐up visits. (a) Custom Restoration Region 36 at BL, (b) Custom Restoration Region 36 at 3Y, (c) Ti‐Base Restoration Regio 36 at BL, (d) Ti‐Base Restoration Regio 36 at 3Y.

### 3.5 Technical Outcomes

No major technical complications—including crown fracture, abutment fracture, or decementation—were observed. According to modified USPHS criteria, one small chipping of the zirconia crown (USPHS Rating B) occurred in the Ti‐Base group at 3 years, corresponding to a technical complication rate of 3.6% in this group (Figure 4). Open proximal contacts were noted at the 3Y‐FU: mesial contacts in one implant in the Custom group and two implants in the Ti‐Base group, and distal contacts in one implant per group. None of these cases required therapeutic intervention, as patients reported no discomfort. The full findings according to USPSH criteria are presented in Table 4. No significant differences were found in USPHS criteria at any time point between the groups (*p* > 0.05).

**FIGURE 4:**
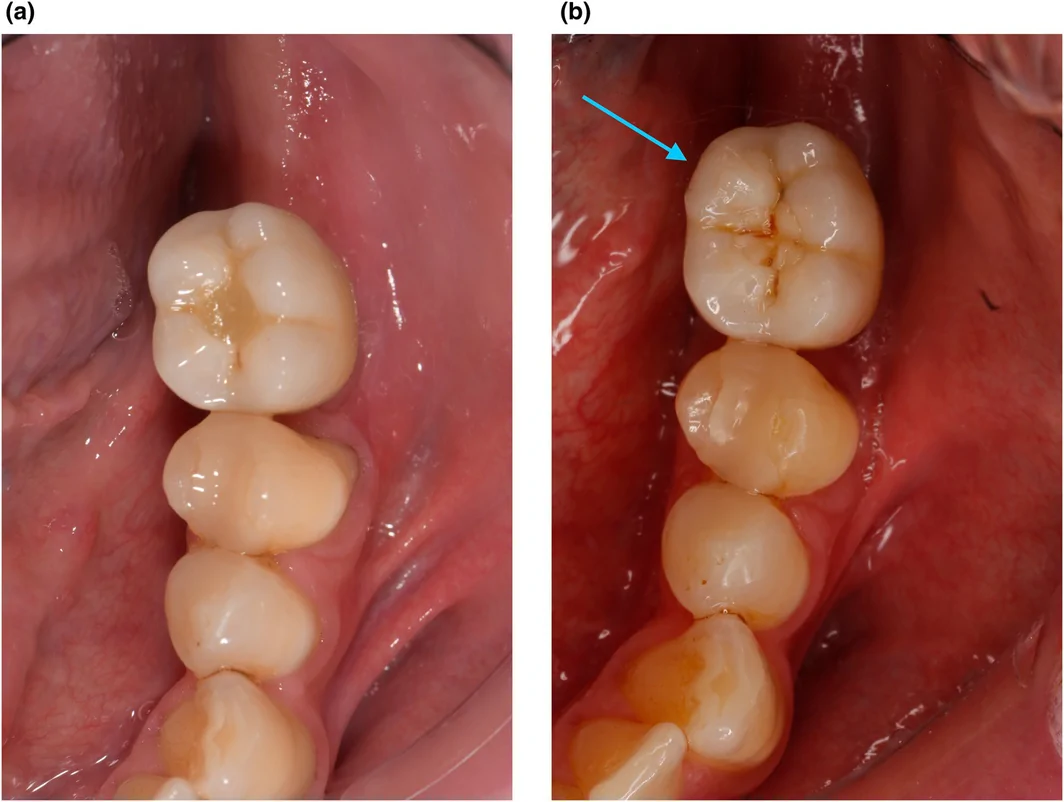
(a) Clinical image at baseline of a Ti‐Base implant crown at region 36. (b) Clinical image on 3‐year follow‐up visit of the same Ti‐Base Implant crown showing chipping of the disto‐lingual cusp (blue arrow).

**TABLE 4:** Clinical evaluation of prosthetic outcomes (excluding major technical complications) at 3‐year follow‐up (3Y‐FU), assessed using modified USPHS criteria. Data are presented as number of cases (n) and percentage (%) within each study group.

Category/Rating	Group	Alpha A *n* (%)	Bravo B *n* (%)	Charlie C *n* (%)	Delta D *n* (%)	Na^a^ *n* (%)
Patient's satisfaction	Ti‐Base	26 (92.9%)	2 (7.1%)	—	—	—
	Custom	23 (100%)	—	—	—	—
Marginal adaptation crown unit	Ti‐Base	27 (96.4%)	1 (3.6%)	—	—	—
	Custom	23 (100%)	—	—	—	—
Anatomical form	Ti‐Base	23 (82.1%)	5 (17.9%)	—	—	—
	Custom	20 (87.0%)	3 (13.0%)	—	—	—
Proximal contact distal	Ti‐Base	15 (53.4%)	4 (14.3%)	1 (3.6%)	—	8 (28.6%)
	Custom	7 (30.4%)	2 (8.7%)	1 (4.3%)	—	13 (56.5%)
Proximal contact mesial	Ti‐Base	20 (71.4%)	5 (17.9%)	2 (7.1%)	—	1 (3.6%)
	Custom	19 (82.6%)	3 (13.0%)	1 (4.3%)	—	—
Occlusion	Ti‐Base	25 (89.3%)	1 (3.6%)	2 (7.1%)	—	—
	Custom	21 (91.3%)	1 (4.3%)	—	—	1 (4.3%)
Occlusal wear	Ti‐Base	16 (57.1%)	11 (39.3%)	1 (3.6%)	—	—
	Custom	15 (65.2%)	8 (34.8%)	—	—	—
Color	Ti‐Base	16 (57.1%)	11 (39.3%)	1 (3.6%)	—	—
	Custom	14 (39.1%)	9 (39.1%)	—	—	—
X‐ray image	Ti‐Base	28 (100%)	—	—	—	—
	Custom	23 (100%)	—	—	—	—

^a^ Na (not applicable): indicates that the information was either missing (e.g., occlusion, occlusal wear) or not present (e.g., absence of neighboring tooth distal or mesial).

### 3.6 Biological Outcomes

Clinical inspection revealed healthy peri‐implant tissues at the 3Y‐FU. Probing pocket depth (PPD) decreased slightly from baseline, with a mean change of −0.50 mm in the Custom group and −0.17 mm in the Ti‐Base group. Ti‐Base crowns showed significantly larger PPD values compared to Custom crowns with a mean difference of −0.6 mm at 1Y‐FU and −0.7 mm at 3Y‐FU (*p* = 0.001) (Table 5). Mucosal recession was minimal, with mean changes from BL to 3Y‐FU of 0.35 mm (SD 0.78) in the Custom group and 0.18 mm (SD 0.94) in the Ti‐Base group (Table 5).

**TABLE 5:** Descriptive data of all biological parameters.

Variable	Time‐point	Group	*n*	Mean	SD	Min	Median	IQR	Max	Mean difference (Custom – Ti‐Base)	95% CI (Custom – Ti‐Base)	*p*
PCR (%)	Baseline	Custom	28	15.0	19.0	0.0	8.0	0.0; 25.0	67.0	−1.0	−10.7 9.3	0.900
		Ti‐Base	30	16.0	19.0	0.0	8.0	0.0; 33.0	67.0			
	1 year	Custom	26	23.0	22.0	0.0	17.0	0.0; 33.0	67.0	2.0	−12.9 16.0	0.787
		Ti‐Base	30	21.0	23.0	0.0	17.0	0.0; 33.0	100.0			
	3 years	Custom	23	19.0	16.0	0.0	17.0	0.0; 33.0	50.0	−5.0	−16.7 6.7	0.658
		Ti‐Base	28	24.0	24.0	0.0	17.0	0.0; 33.0	83.0			
PPD (mm)	Baseline	Custom	28	3.3	0.5	2.7	3.3	3.0; 3.5	4.8	−0.2	−0.5 0.1	0.091
		Ti‐Base	30	3.5	0.5	2.5	3.5	3.2; 3.8	4.5			
	1 year	Custom	26	3.2	0.5	2.3	3.2	3.0; 3.3	4.3	−0.6	−0.9 −0.3	**0.001**
		Ti‐Base	30	3.8	0.7	2.7	3.8	3.2; 4.2	5.3			
	3 years	Custom	23	2.6	0.4	1.7	2.7	2.3; 2.8	3.2	−0.7	−1.1 −0.4	**0.001**
		Ti‐Base	28	3.3	0.8	2.2	3.1	2.8; 3.8	5.3			
BoP (%)	Baseline	Custom	28	32.0	29.0	0.0	33.0	0.0; 50.0	83.0	−4.0	−19.4 12.5	0.702
		Ti‐Base	30	36.0	31.0	0.0	33.0	0.0; 50.0	100.0			
	1 year	Custom	26	44.0	26.0	0.0	42.0	17.0; 67.0	83.0	−8.0	−24.9 7.6	0.318
		Ti‐Base	30	52.0	34.0	0.0	50.0	17.0; 83.0	100.0			
	3 years	Custom	23	28.0	22.0	0.0	33.0	17.0; 50.0	67.0	−6.0	−18.7 7.1	0.454
		Ti‐Base	28	34.0	24.0	0.0	33.0	17.0; 42.0	100.0			
KM (mm)	Baseline	Custom	28	2.6	1.0	1.0	3.0	2.0; 3.0	4.0	−0.5	−1.3 0.2	0.141
		Ti‐Base	30	3.1	1.2	1.0	3.0	2.5; 4.0	5.0			
	1 year	Custom	26	2.6	0.9	1.0	3.0	2.0; 3.0	4.0	−0.4	−1.1 0.3	0.267
		Ti‐Base	30	3.0	1.1	0.5	3.0	3.0; 4.0	5.0			
	3 years	Custom	23	2.5	1.2	0.0	2.0	2.0; 3.0	5.0	−0.3	−1.2 0.6	0.426
		Ti‐Base	28	2.8	1.3	0.0	3.0	2.0; 4.0	5.0			
Crown height (mm)	Baseline	Custom	28	8.5	1.45	6.0	8.0	7.0; 10.0	12.0	−0.2	−1.1 0.7	0.669
		Ti‐Base	30	8.7	1.91	5.0	9.0	7.0; 10.0	13.0			
	1 year	Custom	26	8.5	1.9	5.0	8.0	7.0; 10.0	13.0	−0.2	−1.2 0.9	0.907
		Ti‐Base	30	8.7	2.1	5.0	9.0	7.0; 10.0	13.0			
	3 years	Custom	23	8.8	1.9	5.0	8.0	8.0; 10.0	13.0	−0.2	−1.4 1.1	0.826
		Ti‐Base	28	9.0	2.3	5.0	9.0	7.0; 11.0	13.0			

Abbreviations: 95% CI, 95% confidence interval; BoP, bleeding on probing; crown height, mid buccal distance from cusp to mucosal margin; Custom, monolithic zirconia crown with customized titanium abutment; IQR, interquartile range; KM, keratinized mucosa; Max, maximum; Mean difference, difference between the groups for each parameter at each timepoint; Min, minimum; *n*, number of patients/implants; PCR, plaque control record; PPD, probing pocket depth; SD, standard deviation; Ti‐Base, monolithic zirconia crown with titanium‐base abutment.

The width of keratinized mucosa (KM) remained stable over time, with mean values at 3Y‐FU of 2.5 mm (SD 1.22) for the Custom group and 2.79 mm (SD 1.27) for the Ti‐Base group. Mean reductions in KM from BL to 3Y‐FU were −0.12 mm (SD 0.87) for the Custom group and −0.44 mm (SD 0.98) for the Ti‐Base group (Table 5).

The median plaque index (PI) at 3Y‐FU was 8% in both groups, while the median bleeding on probing (BoP) was 33% in both groups, corresponding to a diagnosis of peri‐implant mucositis in 52.2% (*n* = 12) of Custom group implants and 60.7% (*n* = 17) of Ti‐Base group implants. No cases of peri‐implantitis were diagnosed (Table 5). No statistically significant differences in any of the biological findings were seen between the different time points or between the groups (*p* > 0.05).

### 3.7 Patient Reported Outcome Measures (PROMs)

Throughout the study follow‐up, all patients reported high satisfaction with the treatment. At the BL visit, 25 patients (89.3%) in the Custom group and all the patients in the Ti‐Base group were very satisfied (USPHS Rating A) with the treatment (*p* = 0.068). At 3Y‐FU, all patients (100%, *n* = 23) in the Custom group and 26 patients (92.9%) in the Ti‐Base group continued reporting high satisfaction with their treatment (USPHS Rating A) (*p* = 0.195).

## 4 Discussion

This study evaluated the interim 3‐year outcomes of implant‐supported monolithic zirconia single crowns using standardized titanium base abutments compared with customized titanium abutments, focusing on marginal bone levels, technical and biological outcomes, and patient‐reported outcome measures (PROMs). Both restoration types demonstrated high survival rates and comparable technical and biological performance; no statistically significant differences were seen in any studied parameters (*p* > 0.05). Accordingly, the null hypothesis could be accepted.

The present study demonstrated stable marginal bone levels in both study groups, with median MBL changes ranging from 0.01 to 0.09 mm, indicating similar outcomes for both abutment types. These results are consistent with previous studies reporting short‐ and mid‐term marginal bone level changes for Ti‐Base restorations (Beck et al. 2024; Rathe et al. 2022). For instance, a prospective cohort study of 24 single implants restored with zirconia crowns on Ti‐Base abutments reported a mean MBL change of 0.35 mm over a 7.5‐year follow‐up (Smirani et al. 2024).

Existing clinical evidence has suggested a potential association between the restorative angle of implant‐supported crowns and peri‐implant marginal bone levels, with angles greater than 30° proposed as a risk indicator for peri‐implantitis (Katafuchi et al. 2018; Strauss et al. 2022). Greater angular variations may occur with certain prefabricated implant components, such as Ti‐Base abutments (Strauss et al. 2022). However, the larger marginal bone level changes have been mainly reported during the first year after implant loading (Strauss et al. 2022), and some studies found no correlation between restorative angles and bone levels (Hentenaar et al. 2020; Rathe et al. 2022). The restorative angles were not measured in the present study, but marginal bone level changes remained minimal in both groups over the 3‐year follow‐up.

During the study follow‐up, two implants in the Custom group were lost prior to the 3Y‐FU due to loss of osseointegration. The exact cause of implant loss or potential confounding factors, such as overload, could not be determined. However, the implant loss was not related to peri‐implant health. Previous studies have reported implant survival rates of 89.7%–100% over 10‐year follow‐ups for the same implant type (Di Francesco et al. 2023; Gadzo et al. 2023; Mehrabanian and Dorri 2024). Additionally, a prospective clinical study reported 3‐year survival of 98.3% for implants placed in healed sites (De Bruyn et al. 2013). The findings of the present results are consistent with these previous findings.

In the present study, customized and Ti‐Base implant abutments as well as the monolithic zirconia crowns exhibited a 100% survival rate, with only one technical complication—a small chipping of a zirconia crown in the Ti‐Base group. This is consistent with previous studies, which report very few technical complications for posterior monolithic zirconia crowns on Ti‐Base abutments in prospective and retrospective studies with 3–5 years follow‐up (Beck et al. 2024; Derksen and Wismeijer 2023). A randomized controlled clinical trial with 27 patients comparing conventional and digital workflows reported a success rate of 96.3% (complications rate 3.7%) for monolithic zirconia crowns on titanium base abutments (Beck et al. 2024). The reported complications at 4 years follow‐up included two decementations and one facture of the Ti‐Base abutment. Another RCT with 54 patients reported a 3‐year complication rate of 4.9%, including two cases of screw loosening and one decementation (Derksen and Wismeijer 2023). A recent systematic review summarizing 29 studies concluded that Ti‐Base abutments exhibit high short‐term survival rates (Chantler et al. 2023). However, there is substantial heterogeneity in the geometric design of Ti‐Base abutments, particularly regarding transmucosal height, width, and contour, making direct comparisons between studies challenging. Technical complications are uncommon, with debonding of the suprastructure identified as one of the most frequent issues. Variations in adhesive protocols of Ti‐Base restorations may further contribute to differences in technical outcomes across studies (Hjerppe et al. 2022; Linkevicius et al. 2019; Pitta, Burkhardt, et al. 2021; Wan et al. 2025).

During the 3‐year follow‐up, both restoration groups exhibited stable periodontal parameters, including probing pocket depth, plaque index, and keratinized mucosa, with only minimal mucosal recession. Nevertheless, bleeding on probing (BoP) was elevated at 33%, resulting in a moderate prevalence of peri‐implant mucositis. Interestingly, the proportion of BoP did not correspond with oral hygiene, as the plaque index remained low (8% in both groups). Previous studies on implant crowns with Ti‐Base abutments have reported BoP values ranging from 11% to 27.1% in short‐ and medium‐term follow‐ups (Beck et al. 2024; Joda et al. 2017; Muhlemann et al. 2020; Smirani et al. 2024; Strasding et al. 2023). The present results are therefore at the upper end of this range. However, BoP values may not directly indicate inflammation, as they can be influenced by probing force (trauma) and technique (Monje and Salvi 2024). A clinical study demonstrated that probing forces exceeding 0.15 N can lead to overestimation of peri‐implant mucositis due to false‐positive BoP readings, even in clinically healthy peri‐implant tissues (Gerber et al. 2009). It should be noted that probing the peri‐implant pockets of a molar crown with an internal implant connection might be challenging and may cause local trauma due to the crown and abutment design. This might also be a factor contributing to the high BoP values observed in this study.

The design of customized titanium abutments positions the crown‐abutment interface and cement gap closer to the mucosal margin, whereas in implant restorations based on titanium base abutments, the crown‐abutment margin is situated closer to the marginal bone. The location of the cement margin, as well as the differences in abutment design and transmucosal configuration of the study groups could also influence the biological outcomes. However, no differences were seen between the groups in terms of biological outcomes.

In vitro studies have reported suboptimal marginal adaptation of zirconia crowns on Ti‐Base abutments after artificial aging (Ioannidis et al. 2020; Pitta, Hjerppe, et al. 2021; Schafer et al. 2025). Such discrepancies may facilitate bacterial colonization, especially when crown margins are placed deep submucosally, potentially leading to peri‐implant tissue inflammation and marginal bone loss (Broggini et al. 2003; Cresti et al. 2015). In the present study, one patient in the Ti‐Base group exhibited USPHS Rating B (slight probe catch, but no detectable gap) for marginal adaptation, yet the peri‐implant tissues remained healthy. Overall, no instability of the crown‐abutment interface or cases of peri‐implantitis were observed, and the marginal bone levels remained stable throughout the follow‐up period.

For single implant crown restorations, patient reported outcome measures (PROMs) are commonly investigated in studies comparing conventional and digital fabrication workflows or evaluating aesthetic results (Delize et al. 2019; Joda et al. 2018; Wittneben et al. 2023). The visual analogue scale (VAS) is frequently used to assess patient satisfaction, and generally, high satisfaction levels have been reported (Delize et al. 2019; Joda et al. 2018; Wittneben et al. 2023). In the present study, high patient satisfaction was observed for both restoration types, with 100% of patients in the Custom group and 92.9% of the patients in the Ti‐Base group reporting satisfaction according to interviews using USPHS scale with no significant differences between the groups.

This randomized controlled clinical study provides 3‐year clinical outcomes of monolithic zirconia implant crowns on customized titanium abutments and prefabricated standardized Ti‐Base abutments, presenting unique comparative data that has not been reported in prior randomized studies. However, the present study has some limitations. This randomized controlled clinical study had four study arms, two surgical study arms investigating situations with a buccal dehiscence defect (≤ 5 mm) at the implant placement with and without GBR procedures. Only 16 study sites had a buccal dehiscence at the timepoint of implant placement, resulting in very small numbers in subgroups. Therefore, the study results could not be analyzed according to the four study arms. Results of the surgical sub‐analysis have been published before (Zuercher et al. 2024) and the present manuscript concentrates on the two prosthetic arms of the study, comparing customized titanium abutments and titanium‐base abutments. Due to the COVID‐19 pandemic, some patients missed a single follow‐up visit, and a data imputation method (last observation carried forward, LOCF) was applied for clinical analysis. The use of LOCF was primarily a pragmatic decision, given the disruptions to follow‐up caused by the COVID‐19 pandemic. While participants were retained in the analysis using this approach, it is acknowledged that this method relies on strong assumptions, particularly that outcomes remain stable following the last recorded measurement, which may not accurately reflect the true clinical course. Additionally, some of the patients were lost to follow‐up; seven patients in the Custom group and two patients in the Ti‐Base group. The conventional and digital manufacturing techniques as well as different zirconia materials and abutment designs used for each study group might have an influence on the results, although no differences were seen in the present study. The present study is an interim analysis of an RCT, with the primary outcome being marginal bone level changes at 5 years. The results should be interpreted with caution, since the statistical power is based on the expected outcomes at the 5‐year time point.

## 5 Conclusions

Within the limitations of this interim 3‐year analysis monolithic zirconia single crowns supported by either standardized prefabricated Ti‐base abutments or customized titanium abutments demonstrated stable marginal bone levels, high survival rates, high patient satisfaction and minimal technical complications over the 3‐year follow‐up. This suggests that both treatment modalities may be suitable for posterior single implant restorations.

## Author Contributions

**Sven Mühlemann:** conceptualization, methodology, software, supervision, project administration, funding acquisition, writing – review and editing. **Jenni Hjerppe:** investigation, validation, formal analysis, writing – original draft, writing – review and editing, visualization, data curation. **Ronald E. Jung:** writing – review and editing, supervision, validation, funding acquisition, project administration, resources. **Shunsuke Fukuba:** investigation, visualization, writing – original draft, writing – review and editing. **Stephanie Wenk:** investigation, visualization, writing – original draft, writing – review and editing. **Daniel S. Thoma:** conceptualization, methodology, software, supervision, funding acquisition, project administration, writing – review and editing.

## Funding

Study materials such as implants and abutments (in both test and control groups) were provided by a grant from Dentsply Sirona (I‐AA‐17‐020).

## Conflicts of Interest

The authors declare no conflicts of interest.

## Supporting information


**Data S1:** CONSORT 2010 checklist of information to include when reporting a randomized trial*

## Data Availability

The data that support the findings of this study are available on request from the corresponding author. The data are not publicly available due to privacy or ethical restrictions.

## References

[clr70165-bib-0001] Ainamo, J. , and I. Bay . 1976. “Periodontal Indexes for and in Practice.” Tandlaegebladet 80, no. 5: 149–152. 1078196

[clr70165-bib-0002] Bayne, S. C. , and G. Schmalz . 2005. “Reprinting the Classic Article on USPHS Evaluation Methods for Measuring the Clinical Research Performance of Restorative Materials.” Clinical Oral Investigations 9, no. 4: 209–214. 10.1007/s00784-005-0017-0. 16421996

[clr70165-bib-0003] Beck, F. , L. Zupancic Cepic , S. Lettner , et al. 2024. “Clinical and Radiographic Outcomes of Single Implant‐Supported Zirconia Crowns Following a Digital and Conventional Workflow: Four‐Year Follow‐Up of a Randomized Controlled Clinical Trial.” Journal of Clinical Medicine 13, no. 2: 432. 10.3390/jcm13020432. PMC10816133 38256565

[clr70165-bib-0004] Berglundh, T. , G. Armitage , M. G. Araujo , et al. 2018. “Peri‐Implant Diseases and Conditions: Consensus Report of Workgroup 4 of the 2017 World Workshop on the Classification of Periodontal and Peri‐Implant Diseases and Conditions.” Journal of Clinical Periodontology 45, no. Suppl 20: S286–S291. 10.1111/jcpe.12957. 29926491

[clr70165-bib-0005] Broggini, N. , L. M. McManus , J. S. Hermann , et al. 2003. “Persistent Acute Inflammation at the Implant‐Abutment Interface.” Journal of Dental Research 82, no. 3: 232–237. 10.1177/154405910308200316. 12598555

[clr70165-bib-0006] Buser, D. , S. F. Janner , J. G. Wittneben , U. Bragger , C. A. Ramseier , and G. E. Salvi . 2012. “10‐Year Survival and Success Rates of 511 Titanium Implants With a Sandblasted and Acid‐Etched Surface: A Retrospective Study in 303 Partially Edentulous Patients.” Clinical Implant Dentistry and Related Research 14, no. 6: 839–851. 10.1111/j.1708-8208.2012.00456.x. 22897683

[clr70165-bib-0007] Calderon, U. , S. P. Hicklin , P. Mojon , et al. 2022. “Influence of the Titanium Base Abutment Design on Monolithic Zirconia Multiple‐Unit Implant Fixed Dental Prostheses: A Laboratory Study.” International Journal of Oral and Maxillofacial Implants 37, no. 1: 19–29. 10.11607/jomi.8923. 35235616

[clr70165-bib-0008] Chantler, J. G. M. , C. D. J. Evans , N. U. Zitzmann , and W. Derksen . 2023. “Clinical Performance of Single Implant Prostheses Restored Using Titanium Base Abutments: A Systematic Review and Meta‐Analysis.” Clinical Oral Implants Research 34, no. Suppl 26: 64–85. 10.1111/clr.14128. 37750524

[clr70165-bib-0009] Cresti, S. , A. Itri , A. Rebaudi , A. Diaspro , and M. Salerno . 2015. “Microstructure of Titanium‐Cement‐Lithium Disilicate Interface in CAD‐CAM Dental Implant Crowns: A Three‐Dimensional Profilometric Analysis.” Clinical Implant Dentistry and Related Research 17, no. Suppl 1: e97–e106. 10.1111/cid.12133. 23968260

[clr70165-bib-0010] De Bruyn, H. , F. Raes , L. F. Cooper , et al. 2013. “Three‐Years Clinical Outcome of Immediate Provisionalization of Single Osseospeed(^TM^) Implants in Extraction Sockets and Healed Ridges.” Clinical Oral Implants Research 24, no. 2: 217–223. 10.1111/j.1600-0501.2012.02449.x. 22469026

[clr70165-bib-0011] Delize, V. , A. Bouhy , F. Lambert , and M. Lamy . 2019. “Intrasubject Comparison of Digital vs. Conventional Workflow for Screw‐Retained Single‐Implant Crowns: Prosthodontic and Patient‐Centered Outcomes.” Clinical Oral Implants Research 30, no. 9: 892–902. 10.1111/clr.13494. 31183902

[clr70165-bib-0012] Derksen, W. , and D. Wismeijer . 2023. “Three‐Year Follow‐Up of a Randomized Clinical Trial on Screw‐Retained Monolithic Zirconia Restorations on Ti‐Base Abutments Based on Digital or Conventional Impression Techniques.” International Journal of Prosthodontics 36, no. 4: 410–415. 10.11607/ijp.7891. 37699181

[clr70165-bib-0013] Di Francesco, F. , C. M. Cristache , G. Minervini , E. A. Cafferata , and A. Lanza . 2023. “Survival and Mechanical Complications of Single‐ and Multiple‐Unit Cement‐Retained Posterior Implant‐Supported Restorations With Custom CAD/CAM Atlantis Titanium Abutments: An up to 10‐Year Retrospective Analysis.” International Journal of Oral Implantology (Berlin) 16, no. 4: 315–324. 37994819

[clr70165-bib-0014] Gadzo, N. , A. Ioannidis , N. Naenni , J. Husler , R. E. Jung , and D. S. Thoma . 2023. “Survival and Complication Rates of Two Dental Implant Systems Supporting Fixed Restorations: 10‐Year Data of a Randomized Controlled Clinical Study.” Clinical Oral Investigations 27, no. 12: 7327–7336. 10.1007/s00784-023-05323-5. PMC10713755 37906305

[clr70165-bib-0015] Gerber, J. A. , W. C. Tan , T. E. Balmer , G. E. Salvi , and N. P. Lang . 2009. “Bleeding on Probing and Pocket Probing Depth in Relation to Probing Pressure and Mucosal Health Around Oral Implants.” Clinical Oral Implants Research 20, no. 1: 75–78. 10.1111/j.1600-0501.2008.01601.x. 19126110

[clr70165-bib-0016] Hentenaar, D. F. , Y. C. De Waal , A. J. Van Winkelhoff , G. M. Raghoebar , and H. J. Meijer . 2020. “Influence of Cervical Crown Contour on Marginal Bone Loss Around Platform‐Switched Bone‐Level Implants: A 5‐Year Cross‐Sectional Study.” International Journal of Prosthodontics 33, no. 4: 373–379. 10.11607/ijp.6365. 32639696

[clr70165-bib-0017] Herrera, D. , T. Berglundh , F. Schwarz , et al. 2023. “Prevention and Treatment of Peri‐Implant Diseases‐The EFP S3 Level Clinical Practice Guideline.” Journal of Clinical Periodontology 50, no. Suppl 26: 4–76. 10.1111/jcpe.13823. 37271498

[clr70165-bib-0018] Hjerppe, J. , R. E. Jung , C. H. Hammerle , M. Ozcan , and S. Muhlemann . 2022. “Mechanical Stability of Fully Personalized, Abutment‐Free Zirconia Implant Crowns on a Novel Implant‐Crown Interface.” Journal of Dentistry 121: 104121. 10.1016/j.jdent.2022.104121. 35395344

[clr70165-bib-0019] Humm, V. L. , I. Sailer , D. S. Thoma , C. H. F. Hammerle , R. E. Jung , and A. Zembic . 2023. “13‐Year Follow‐Up of a Randomized Controlled Study on Zirconia and Titanium Abutments.” Clinical Oral Implants Research 34, no. 9: 911–919. 10.1111/clr.14117. 37340530

[clr70165-bib-0020] Ioannidis, A. , A. Gil , C. H. Hammerle , R. E. Jung , S. Zinelis , and G. Eliades . 2020. “Effect of Thermomechanical Loading on the Cementation Interface of Implant‐Supported CAD/CAM Crowns Luted to Titanium Abutments.” International Journal of Prosthodontics 33, no. 6: 656–662. 10.11607/ijp.6709. 33284908

[clr70165-bib-0021] Joda, T. , M. Ferrari , and U. Bragger . 2017. “Monolithic Implant‐Supported Lithium Disilicate (LS2) Crowns in a Complete Digital Workflow: A Prospective Clinical Trial With a 2‐Year Follow‐Up.” Clinical Implant Dentistry and Related Research 19, no. 3: 505–511. 10.1111/cid.12472. 28093860

[clr70165-bib-0022] Joda, T. , M. Ferrari , U. Bragger , and N. U. Zitzmann . 2018. “Patient Reported Outcome Measures (PROMs) of Posterior Single‐Implant Crowns Using Digital Workflows: A Randomized Controlled Trial With a Three‐Year Follow‐Up.” Clinical Oral Implants Research 29, no. 9: 954–961. 10.1111/clr.13360. 30144159

[clr70165-bib-0023] Joda, T. , J. Katsoulis , and U. Bragger . 2016. “Clinical Fitting and Adjustment Time for Implant‐Supported Crowns Comparing Digital and Conventional Workflows.” Clinical Implant Dentistry and Related Research 18, no. 5: 946–954. 10.1111/cid.12377. 26395013

[clr70165-bib-0024] Jung, R. E. , A. Zembic , B. E. Pjetursson , M. Zwahlen , and D. S. Thoma . 2012. “Systematic Review of the Survival Rate and the Incidence of Biological, Technical, and Aesthetic Complications of Single Crowns on Implants Reported in Longitudinal Studies With a Mean Follow‐Up of 5 Years.” Clinical Oral Implants Research 23, no. Suppl 6: 2–21. 10.1111/j.1600-0501.2012.02547.x. 23062124

[clr70165-bib-0025] Katafuchi, M. , B. F. Weinstein , B. G. Leroux , Y. W. Chen , and D. M. Daubert . 2018. “Restoration Contour Is a Risk Indicator for Peri‐Implantitis: A Cross‐Sectional Radiographic Analysis.” Journal of Clinical Periodontology 45, no. 2: 225–232. 10.1111/jcpe.12829. 28985447

[clr70165-bib-0026] Kraus, R. D. , J. Hjerppe , N. Naenni , M. Balmer , R. E. Jung , and D. S. Thoma . 2024. “A 7.5‐Year Randomized Controlled Clinical Study Comparing Cemented and Screw‐Retained One‐Piece Zirconia‐Based Implant‐Supported Single Crowns.” Clinical Oral Implants Research 35, no. 12: 1669–1675. 10.1111/clr.14346. PMC11629449 39172056

[clr70165-bib-0027] Linkevicius, T. , A. Caplikas , I. Dumbryte , L. Linkeviciene , and O. Svediene . 2019. “Retention of Zirconia Copings Over Smooth and Airborne‐Particle‐Abraded Titanium Bases With Different Resin Cements.” Journal of Prosthetic Dentistry 121, no. 6: 949–954. 10.1016/j.prosdent.2018.08.012. 30711294

[clr70165-bib-0028] Marchack, C. B. 1996. “A Custom Titanium Abutment for the Anterior Single‐Tooth Implant.” Journal of Prosthetic Dentistry 76, no. 3: 288–291. 10.1016/s0022-3913(96)90173-0. 8887802

[clr70165-bib-0029] Mehrabanian, M. , and M. Dorri . 2024. “Long‐Term Survival of Astra Tech vs Straumann Dental Implants and Restorations.” Evidence‐Based Dentistry 25, no. 1: 33–34. 10.1038/s41432-024-00974-z. 38263242

[clr70165-bib-0030] Moher, D. , S. Hopewell , K. F. Schulz , et al. 2010. “CONSORT 2010 Explanation and Elaboration: Updated Guidelines for Reporting Parallel Group Randomised Trials.” BMJ 340: c869. 10.1136/bmj.c869. PMC2844943 20332511

[clr70165-bib-0031] Monje, A. , and G. E. Salvi . 2024. “Diagnostic Methods/Parameters to Monitor Peri‐Implant Conditions.” Periodontology 2000 95, no. 1: 20–39. 10.1111/prd.12584. 38923148

[clr70165-bib-0032] Muhlemann, S. , R. D. Kraus , C. H. F. Hammerle , and D. S. Thoma . 2018. “Is the Use of Digital Technologies for the Fabrication of Implant‐Supported Reconstructions More Efficient and/or More Effective Than Conventional Techniques: A Systematic Review.” Clinical Oral Implants Research 29, no. Suppl 18: 184–195. 10.1111/clr.13300. 30306680

[clr70165-bib-0033] Muhlemann, S. , T. Lakha , R. E. Jung , C. H. F. Hammerle , and G. I. Benic . 2020. “Prosthetic Outcomes and Clinical Performance of CAD‐CAM Monolithic Zirconia Versus Porcelain‐Fused‐To‐Metal Implant Crowns in the Molar Region: 1‐Year Results of a RCT.” Clinical Oral Implants Research 31, no. 9: 856–864. 10.1111/clr.13631. 32562324

[clr70165-bib-0034] Muhlemann, S. , S. T. Lamperti , L. Stucki , C. H. F. Hammerle , and D. S. Thoma . 2022. “Time Efficiency and Efficacy of a Centralized Computer‐Aided‐Design/Computer‐Aided‐Manufacturing Workflow for Implant Crown Fabrication: A Prospective Controlled Clinical Study.” Journal of Dentistry 127: 104332. 10.1016/j.jdent.2022.104332. 36252858

[clr70165-bib-0035] Nascimento, C. , L. N. Ikeda , M. S. Pita , et al. 2015. “Marginal Fit and Microbial Leakage Along the Implant‐Abutment Interface of Fixed Partial Prostheses: An in Vitro Analysis Using Checkerboard DNA‐DNA Hybridization.” Journal of Prosthetic Dentistry 114, no. 6: 831–838. 10.1016/j.prosdent.2015.05.009. 26359546

[clr70165-bib-0036] O'Leary, T. J. , R. B. Drake , and J. E. Naylor . 1972. “The plaque control record.” Journal of Periodontology 43, no. 1: 38. 10.1902/jop.1972.43.1.38. 4500182

[clr70165-bib-0037] Palmer, R. M. , P. J. Palmer , and B. J. Smith . 2000. “A 5‐Year Prospective Study of Astra Single Tooth Implants.” Clinical Oral Implants Research 11, no. 2: 179–182. 10.1111/j.1600-0501.2000.tb00012.x. 11217232

[clr70165-bib-0038] Pitta, J. , F. Burkhardt , M. Mekki , V. Fehmer , P. Mojon , and I. Sailer . 2021. “Effect of Airborne‐Particle Abrasion of a Titanium Base Abutment on the Stability of the Bonded Interface and Retention Forces of Crowns After Artificial Aging.” Journal of Prosthetic Dentistry 126, no. 2: 214–221. 10.1016/j.prosdent.2020.06.013. 32921422

[clr70165-bib-0039] Pitta, J. , J. Hjerppe , F. Burkhardt , V. Fehmer , P. Mojon , and I. Sailer . 2021. “Mechanical Stability and Technical Outcomes of Monolithic CAD/CAM Fabricated Abutment‐Crowns Supported by Titanium Bases: An in Vitro Study.” Clinical Oral Implants Research 32, no. 2: 222–232. 10.1111/clr.13693. 33258267

[clr70165-bib-0040] Pitta, J. , C. Zarauz , B. Pjetursson , I. Sailer , X. Liu , and G. Pradies . 2020. “A Systematic Review and Meta‐Analysis of the Influence of Abutment Material on Peri‐Implant Soft Tissue Color Measured Using Spectrophotometry.” International Journal of Prosthodontics 33, no. 1: 39–47. 10.11607/ijp.6393. 31860912

[clr70165-bib-0041] Pjetursson, B. E. , N. A. Valente , M. Strasding , M. Zwahlen , S. Liu , and I. Sailer . 2018. “A Systematic Review of the Survival and Complication Rates of Zirconia‐Ceramic and Metal‐Ceramic Single Crowns.” Clinical Oral Implants Research 29, no. Suppl 16: 199–214. 10.1111/clr.13306. 30328190

[clr70165-bib-0042] Pjetursson, B. E. , C. Zarauz , M. Strasding , I. Sailer , M. Zwahlen , and A. Zembic . 2018. “A Systematic Review of the Influence of the Implant‐Abutment Connection on the Clinical Outcomes of Ceramic and Metal Implant Abutments Supporting Fixed Implant Reconstructions.” Clinical Oral Implants Research 29, no. Suppl 18: 160–183. 10.1111/clr.13362. 30306682

[clr70165-bib-0043] Rathe, F. , R. Junker , C. Heumann , et al. 2022. “Long‐Term Effects of Titanium‐Base Abutments on Peri‐Implant Health: A 5‐Year Randomised Controlled Trial.” International Journal of Oral Implantology (Berlin) 15, no. 2: 167–179. 35546725

[clr70165-bib-0044] Sailer, I. , A. Zembic , R. E. Jung , D. Siegenthaler , C. Holderegger , and C. H. Hammerle . 2009. “Randomized Controlled Clinical Trial of Customized Zirconia and Titanium Implant Abutments for Canine and Posterior Single‐Tooth Implant Reconstructions: Preliminary Results at 1 Year of Function.” Clinical Oral Implants Research 20, no. 3: 219–225. 10.1111/j.1600-0501.2008.01636.x. 19397632

[clr70165-bib-0045] Sasada, Y. , and D. L. Cochran . 2017. “Implant‐Abutment Connections: A Review of Biologic Consequences and Peri‐Implantitis Implications.” International Journal of Oral and Maxillofacial Implants 32, no. 6: 1296–1307. 10.11607/jomi.5732. 29140374

[clr70165-bib-0046] Schafer, T. , K. J. Matzener , R. E. Jung , M. Ozcan , and J. Hjerppe . 2025. “Load‐Bearing Capacity of Screw‐Retained Fixed Dental Prostheses Made of Monolithic Zirconia on Different Abutment Designs and Abutment‐Free Implant Connection.” Journal of Dentistry 153: 105561. 10.1016/j.jdent.2025.105561. 39778736

[clr70165-bib-0047] Shao, J. , and B. Zhong . 2003. “Last Observation Carry‐Forward and Last Observation Analysis.” Statistics in Medicine 22, no. 15: 2429–2441. 10.1002/sim.1519. 12872300

[clr70165-bib-0048] Smirani, R. , J. G. Chantler , J. Endres , et al. 2024. “Clinical Outcomes of Single Implant Supported Crowns Utilising the Titanium Base Abutment: A 7.5‐Year Prospective Cohort Study.” Journal of Dentistry 149: 105306. 10.1016/j.jdent.2024.105306. 39154834

[clr70165-bib-0049] Strasding, M. , S. P. Hicklin , A. Todorovic , V. Fehmer , P. Mojon , and I. Sailer . 2023. “A Multicenter Randomized Controlled Clinical Pilot Study of Buccally Micro‐Veneered Lithium‐Disilicate and Zirconia Crowns Supported by Titanium Base Abutments: 1‐Year Outcomes.” Clinical Oral Implants Research 34, no. 1: 56–65. 10.1111/clr.14018. PMC10098475 36321877

[clr70165-bib-0050] Strauss, F. J. , M. Siegenthaler , C. H. F. Hammerle , I. Sailer , R. E. Jung , and D. S. Thoma . 2022. “Restorative Angle of Zirconia Restorations Cemented on Non‐Original Titanium Bases Influences the Initial Marginal Bone Loss: 5‐Year Results of a Prospective Cohort Study.” Clinical Oral Implants Research 33, no. 7: 745–756. 10.1111/clr.13954. PMC9543966 35570366

[clr70165-bib-0051] Teixeira, W. , R. F. Ribeiro , S. Sato , and V. Pedrazzi . 2011. “Microleakage Into and From Two‐Stage Implants: An in Vitro Comparative Study.” International Journal of Oral and Maxillofacial Implants 26, no. 1: 56–62. 21365038

[clr70165-bib-0052] Tonetti, M. S. , M. Sanz , G. Avila‐Ortiz , et al. 2023. “Relevant Domains, Core Outcome Sets and Measurements for Implant Dentistry Clinical Trials: The Implant Dentistry Core Outcome Set and Measurement (ID‐COSM) International Consensus Report.” Journal of Clinical Periodontology 50, no. Suppl 25: 5–21. 10.1111/jcpe.13808. 37143289

[clr70165-bib-0053] Wan, Q. , D. H. Lee , R. J. Y. Kim , I. L. Yeo , J. H. Lee , and I. Sailer . 2025. “Pull‐Out Force and Fracture Load of Fully Sintered Zirconia Hybrid Abutment‐Crowns on Ti‐Base Abutments: Surface Treatment Effects and Material Comparison.” Journal of Dentistry 163: 106134. 10.1016/j.jdent.2025.106134. 41015219

[clr70165-bib-0054] Wittneben, J. G. , B. Yilmaz , D. Wismeijer , S. Shahdad , U. Bragger , and S. Abou‐Ayash . 2023. “Patient‐Reported Outcome Measures Focusing on the Esthetics of Implant‐Compared to Tooth‐Supported Single Crowns‐A Systematic Review and Meta‐Analysis.” Journal of Esthetic and Restorative Dentistry 35, no. 4: 632–645. 10.1111/jerd.12983. 36479822

[clr70165-bib-0055] Zhang, M. , D. K. L. Ho , G. Pelekos , and M. R. Fok . 2024. “Clinical Performance of Implant‐Supported Single Hybrid Abutment Crown Restoration: A Systematic Review and Meta‐Analysis.” Journal of Prosthodontic Research 68, no. 1: 63–77. 10.2186/jpr.JPR_D_22_00279. 37316260

[clr70165-bib-0056] Zuercher, A. N. , S. Muhlemann , E. Ruales‐Carrera , J. Hjerppe , R. E. Jung , and D. S. Thoma . 2024. “Comparing Small Buccal Dehiscence Defects Treated With or Without Guided Bone Regeneration. A Sub‐Analysis of a RCT.” International Journal of Periodontics and Restorative Dentistry 45, no. 6: 772–783. 10.11607/prd.7138. 39453621

[clr70165-bib-0057] Zumstein, K. , T. Waller , C. H. F. Hammerle , R. E. Jung , G. Benic , and S. Muhlemann . 2023. “Clinical Performance of Monolithic Zirconia Crowns on Titanium‐Zirconium Reduced‐Diameter Implants in the Molar Area: Interim Data at Three Years of a Randomized Controlled Trial.” Clinical Oral Implants Research 34, no. 12: 1354–1362. 10.1111/clr.14179. 37668109

